# Financial well-being advice delivered within the context of social prescribing in the UK and the Republic of Ireland

**DOI:** 10.3389/fpubh.2026.1789734

**Published:** 2026-05-29

**Authors:** Simon Newstead, Amrita Jesurasa, Bethan Jenkins, Teresa Filipponi, Edward Oloidi, Abraham Makanjuola, Carolyn Wallace

**Affiliations:** 1 Faculty of Life Sciences and Education, University of South Wales, Treforest, United Kingdom; 2 Wales School for Social Prescribing Research, Treforest, United Kingdom; 3 Primary Care Division, Health and Wellbeing Directorate, Public Health Wales, Cardiff, United Kingdom

**Keywords:** debt, financial well-being, income maximisation, scoping review, social prescribing, social welfare, welfare benefit

## Abstract

**Introduction:**

The disparity between income and rising prices has pushed many households across the UK into poverty. Social prescribing has the potential to support individuals to improve their financial well-being and address socioeconomic factors that are negatively impacting their health and well-being, an approach that is supported by the National Framework for Social Prescribing. However, little is currently known about the provision of such support within the UK and the Republic of Ireland.

**Methods:**

A scoping review was conducted to explore financial well-being advice and support, delivered within the context of social prescribing in the UK and the Republic of Ireland. Keywords were combined into search strings using Boolean operators to search five electronic databases between 04/02/2025 and 10/02/25.

**Results:**

The full text of 126 articles were screened, resulting in the inclusion of 28 articles in the review (15 x peer-reviewed articles and 13 x grey literature articles).

**Discussion:**

From the literature, we identified three models of financial well-being advice and support within social prescribing: a social prescribing practitioner-led model, a specialist advisor-led model, and a wraparound model. Most articles related to financial well-being advice and support targeted at welfare benefits, debt and legal issues, delivered by specialist advisors co-located in healthcare settings. The delivery of targeted support in this manner was reported as having a number of positive outcomes for patients and healthcare staff, and several strategies were identified to increase the efficacy of such support. Few articles reported on the social prescribing practitioner-led model of delivery of financial well-being advice and support. For those that did, direct support was provided out of necessity due to a lack of availability of specialist advisors in the community, which negatively impacted capacity. While there is potential for social prescribing to play a more integral role in the delivery of financial well-being advice and support and support, further research is required to ensure the efficacious employment of funding and delivery, which may differ across nations and regions.

## Introduction

The wider determinants of health encompass a diverse range of social, economic, environmental, and structural factors that can both directly and indirectly influence health, well-being, and health inequalities (1). Income is one such key determinant of health and well-being (2), impacting the extent to which a person can invest in the basics for a healthy life, such as food and quality housing. Consequently, lower income is directly correlated with poorer mental health (3), and severe debt is related to worse physical and mental health (4, 5), while increased income has been shown to exert a positive impact on general well-being (3).

From November 2021 to June 2024, adults in Britain reported increases in their cost of living compared to the previous month (6). This disparity between income and rising prices is referred to as the ‘cost of living crisis’ and has pushed many households across the UK into poverty. According to the 2021 UK Adult Financial well-being survey: 53% of people struggle to keep up, or are falling behind or have fallen behind with their bills; 29% would be unable to pay an unexpected bill of £300 from spare money or affordable borrowing; 58% do not have a plan for their finances in retirement; and 47% do not feel confident managing their money (7). Such figures highlight the potential threat of the cost-of-living crises on mental and physical health and well-being and the importance of supporting individuals to achieve financial well-being.

Financial well-being is a broad, holistic concept. It goes beyond income to assess an individual’s overall economic health and security, accounting for the management of income and the individual’s sense of control over meeting both short-term and long-term financial needs (8). The Money and Pension Service (9) states that individuals who have a positive financial well-being are financially resilient, confident, and empowered, breaking financial well-being down into five key areas: Receiving a meaningful financial education; Saving regularly; Using credit for everyday essentials; Accessing debt advice; and Planning for and in later life.

One possible means of supporting individuals to improve their financial well-being is to improve access to financial well-being advice and support (FWA/S) through social prescribing; A person-centred approach to connecting people to local community assets (10, 11). The National Framework for Social Prescribing (NFfSP) (11) positions social prescribing as an approach for improving individual health and well-being, reducing inequalities, addressing the influence of social determinants of health, and supporting recovery from the impacts of COVID-19. Strengthening connections to community resources is seen as a key route to achieving these aims. The NFfSP explicitly identifies money worries, debt, and poverty as core well-being issues that social prescribing should seek to address, and highlights that linking people to community assets offering financial and housing advice can help alleviate financial pressures (11).

Additional support for this approach can be found in a Public Health Lens report (12) and guidance on how primary care networks can embed financial support within social prescribing practices (13). The report argues that social prescribing has an important role in mitigating the negative impacts of the cost-of-living crisis, suggesting that social prescribing can contribute through immediate, short-term, and long-term actions across several policy areas, including income and debt (via access to financial well-being advice services), mental health and well-being, and wider health and care support (12). The guidance suggests that including support for money issues within the social prescribing offer could help to prevent ill health across primary care neighbourhoods, support the needs of the existing population within primary care, and reduce inequality faced by people in the more deprived parts of the community (13).

In an earlier discussion paper, Public Health Wales examined the value of advisory services, such as the Citizens Advice Bureau, for health and the wider economy (14). Public Health Wales also noted that evidence on the direct health and financial outcomes linked to the provision of advice services remains limited, recommending further research, particularly within the Welsh context (14). They suggested that: (1) Advisory services could help to reduce pressures on healthcare services and the burden on primary care practitioners who are often people’s ‘first port of call’, by providing support on various issues which may be impacting well-being: and (2) That the provision of advisory services that offer support with debt, personal finance, housing and employment, within healthcare settings, could be particularly beneficial.

There appears to be potential and enthusiasm for social prescribing to contribute to the alleviation of socioeconomic factors that negatively impact health and well-being. Several reviews have previously examined FWA/S interventions [e.g., (15–17)], but little is currently known about the provision of such support within the UK and the Republic of Ireland (RoI), in the context of social prescribing. The Wales School for Social Prescribing Research (WSSPR) and Public Health Wales conducted collaborative research to address this gap.

## Method

A scoping review was conducted to identify the extent and nature of available evidence relating to FWA/S in the context of social prescribing within the UK and RoI, as well as the gaps in knowledge within the field (18, 19). The protocol was based on the scoping review methodological framework proposed by Arksey and O’Malley (18), which outlines a five-stage process: Identifying the research question; Identification of relevant literature; Selection of literature; Charting the literature and data; and Collating, summarising, and reporting the results. This was an iterative process which required reflexive engagement, along with repetition of many of the stages to ensure a comprehensive review of the relevant literature. As is common with scoping reviews, the protocol was refined as familiarity and appreciation of the breadth of material were gained.

### The research question

The research question was identified as: “What does the literature tell us about financial well-being advice and support (FWA/S) delivered within the context of social prescribing in the UK and RoI?”

### Identification of relevant literature

Requests for potentially relevant documentation were made to WSSPR and PHW staff and stakeholders from associated groups. Preliminary database searches were conducted to familiarise ourselves with the potential scope of the literature, associated terminology and to help inform inclusion/exclusion criteria (AM). Potentially relevant documents identified during the preliminary searches and as a result of staff/stakeholder requests were retained and incorporated into the final body of documentation that underwent full-text screening.

#### Search strategy

Keywords were combined into search strings using Boolean operators. Five electronic databases (ASSIA Embase CINAHL Proquest Central Sociology) were searched between 4th February 2025–10th February 2025 using the combined search strings A + C and B + C (Table 1). All searches were examined in their entirety. Snowball searches of citations from relevant literature were conducted to identify additional potentially relevant literature. In addition, related review articles that were identified during the scoping process were examined for potentially relevant literature which was subsequently sourced and screened for inclusion in the review.

**Table 1:** Database search strings.

Search string description	Search string
A: Terms related to interventions that fall under the umbrella of ‘social prescribing’.	“Community connect*” OR “community coordinat*” OR “community referral” OR “community link*” OR “community navigate*” OR “linking scheme” OR “linking service” OR “link navigat*” OR “non-clinical” OR “non-medical” OR “non-pharmaceutical” OR “social prescri*” OR “social referral” OR “supported referral”
B: Terms related to roles that fall under the umbrella of ‘social prescribing practitioner’ (but are not captured by A)	“Advisor” OR “community health champion” OR “facilitator” OR “health broker” OR “health connector” OR “link co-ordinator” OR “link worker” OR “local NEAR coordinator” OR “project worker” OR “support worker” OR “well-being NEAR coordinator”
C: Terms related to ‘financial well-being advice’	“Asset” OR “budget*” OR “debt” OR “financ*” OR “food” OR “hous*” OR “income” OR “legal” OR “poverty” OR “tenancy” OR “welfare”

#### Eligibility criteria

**Figure 1:**
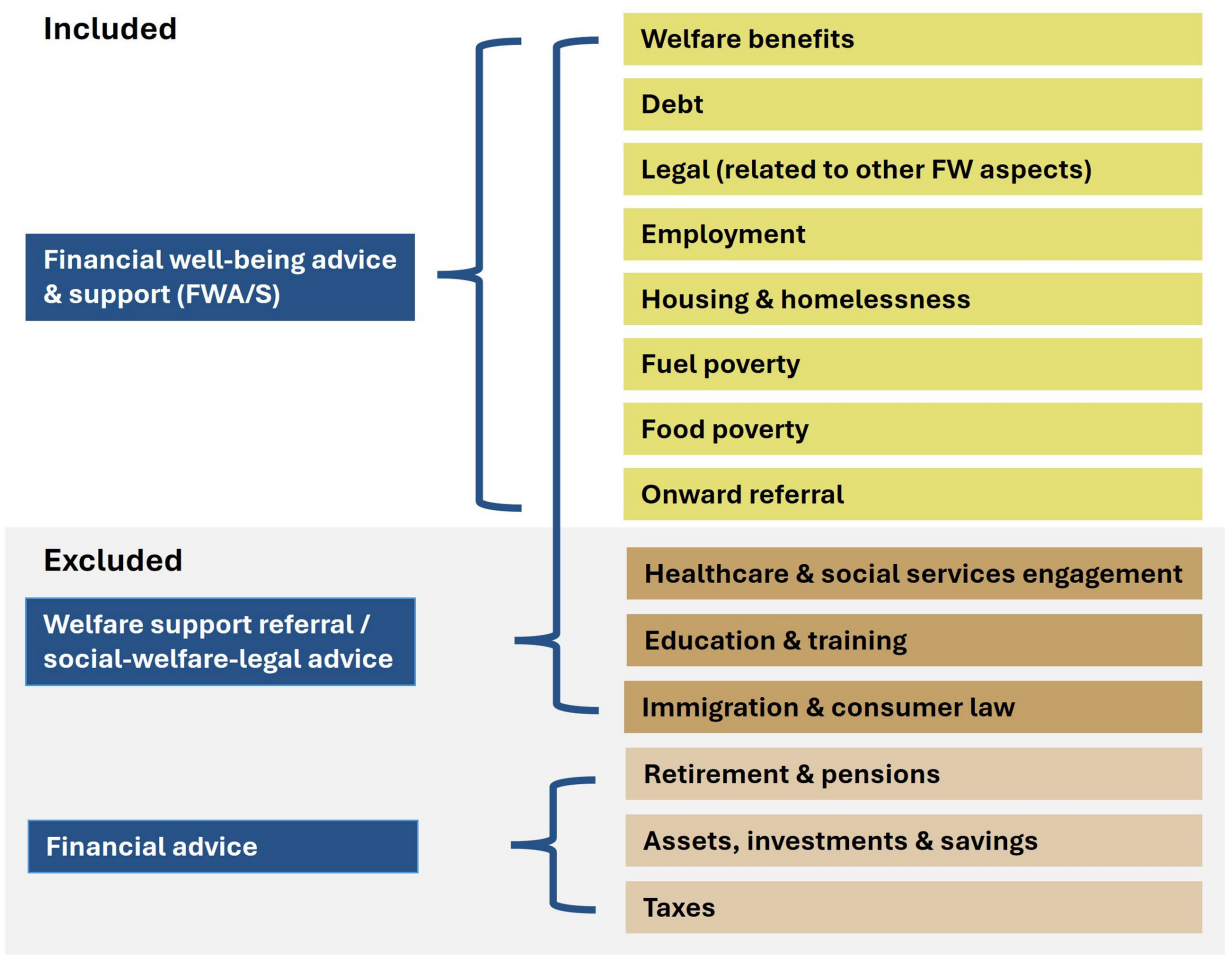
The elements of financial well-being advice and support (FWA/S).

Inclusion criteria are outlined in Table 2. The definition of FWA/S, outlined in the glossary and depicted in Figure 1, was guided by the descriptions of welfare support referral in the glossary of terms for social prescribing and the description of social-welfare-legal advice in the guidance by the National Academy for Social Prescribing (NASP) and Money and Pensions Services (MaPS) (13). FWA/S was defined as the provision of advice and/or support with welfare benefits, debt management, fuel poverty, food poverty, employment, tenancy issues and avoiding homelessness, and legal issues associated with the aforementioned aspects of FWA/S. Financial advice, which includes advice on aspects such as investments, savings, and pensions was considered to be distinctly different from financial well-being (13, 20), as were aspects of welfare support referral and social-welfare-legal advice that were associated with immigration and consumer law, education or training, access to healthcare, and engagement with interventions from social services (13). While it is recognised that gambling and other factors will also affect financial well-being, interventions related to addressing these were not included in the scope of the review.

**Table 2:** Inclusion criteria.

Category	Criteria
Population	Individuals aged ≥16 years old
Intervention	Interventions that 1) fall within the broader context of social prescribing AND 2) include an element of FWA/S with one or more of: Welfare benefits Debt Legal issues Housing/homelessness Employment Fuel poverty Food poverty
Primary outcome	Any reported outcomes related to FWA/S (as outlined above).
Secondary outcomes	Any reported outcomes related to broader health and social, physical and mental well-being.
Timeframe	2004—Onwards
Language	English
Countries	England, Northern Ireland, Scotland, Republic of Ireland, UK, Wales
Evidence type	Peer-reviewed and grey literature: studies, reports, lay summaries

#### Selection of literature

Results from the database searches were imported to www.rayyan.ai. After removing duplicates, SN screened the titles and abstracts to identify potentially relevant literature. As a quality control measure, CW and BJ provided a second screening on 20% of the documents. Where there was a conflict of opinion, the steering group were consulted. Full-text screening was then conducted by SN.

#### Charting the literature and data

Data from the selected literature for inclusion in the review were extracted and input into a spreadsheet (SN). In line with the suggestions of Levac (21), this was an iterative process that required repeated additions and updates to the table as familiarity with the literature was gained. To ensure accuracy, the extracted data was independently reviewed and cross-referenced with the included articles (TF and EO). Perceived omissions of relevant data and/or incorrect entries were highlighted and discussed (SN, TF and EO), and where necessary, amended. The type(s) of FWA/S provided were charted, along with the location in which the service was delivered, the organisation responsible for its delivery, the referral process into the intervention, and any eligibility criteria.

### Categorisation of FWA/S

**Figure 2:**
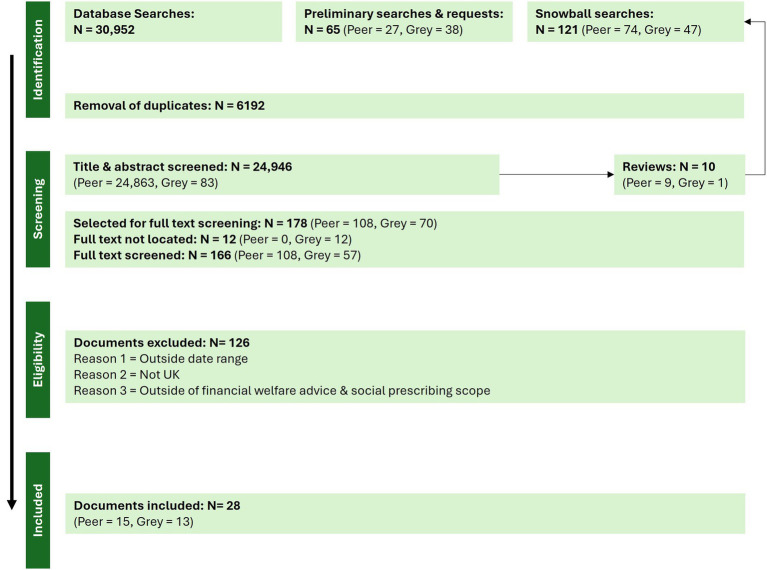
PRISMA diagram for scoping review.

Articles were categorised by the aspects of FWA/S described in the articles (Figure 2 and Supplementary Table 1). Categorisation included: (1) The primary form(s) of FWA/S and/ or onward referral that are described, i.e., the target of the intervention; and (2) The secondary form(s) of FWA/S and/or onward referral that are described.

#### Collating, summarising, and reporting the results

Data synthesis followed the process for analysing, reporting and applying meaning to the results of a scoping review, as outlined by Levac (21). This included a numerical summary and a narrative synthesis to help identify the implications of the review findings for policy, practice, and/or research.

## Results

Twenty-eight articles were included in the scoping review: 15 peer-reviewed articles [1 x realist evaluation (22), 1 x SROI evaluation (23), 13 x studies/service evaluations (24–36)] and 13 grey literature articles 1x case study (37), 1 x mapping study (38), and 11 x report (39–49) (Figure 2 and Supplementary Table 1). One third of the articles were published within the last 5 years, i.e., in or after 2020, which may reflect an increase in interest in this area. The full text of 11 grey literature articles, all published prior to 2015, could not be located.

There was a large qualitative element in many of the articles examined. Interpretation of findings and direct comparisons between studies were made challenging by the diversity of measures employed (Table 3). Eight articles reported using non-standardised questionnaires or surveys in their assessment of intervention efficacy (24, 33, 36, 43–46, 49), and there was little consistency in the use of standardised measures. Given the focus of the articles, surprisingly few articles reported collecting data related to patients/service users’ financial situations. Relatively few studies used standardised measures. Even fewer employed measures that specifically examined changes in physical health (33, 39, 44), although some did use measures that examined aspects of it (34–36, 46). Six articles did not specify outcome or assessment measures (22, 28, 38, 40, 47, 48).

**Table 3:** Outcome and assessment measures identified in the review.

Outcome/assessment measure	# of articles	References
Interviews (patients/service users)	9	(22, 24, 26, 27, 32, 35, 42, 43, 46, 49)
Interviews (HC staff/ advisors/SPPs)	9	(24, 25, 27, 30, 31, 34, 35, 46, 49)
Focus groups	1	(34)
EQ5D	2	(33, 44)
EQ5D-35	1	(39)
Wellbeing (ONS4)	1	(41)
SWEMWBS	2	(23, 36)
State Trait Anxiety Index (STAI-6)	1	(33)
SF-12	1	(46)
GHQ-12	1	(36)
Wellbeing star	2	(34, 35)
Referral data	3	(24, 41, 49)
Service costs	2	(23, 37)
Financial and benefits/grants data	5	(27, 29, 32, 36, 49)
Questionnaires and survey	8	(24, 33, 36, 43–46, 49)
Home temperature	1	(43)
Support provided	1	(24)
Hospital admission data	1	(25)
Housing/homelessness status	1	(25)
CAB outcome data	1	(39)
Medical centre data	1	(30)
None specified	6	(22, 28, 38, 40, 47, 48)

### Categorisation of FWA/S

Articles were categorised by the primary and secondary form(s) of FWA/S support and/ or onward referral that they described (see Supplementary Table 1). Categorisation was at times challenging. While secondary forms of support were often reported, it was not always possible to determine the extent of that support (i.e., signposting, referral, guidance, advice or practical support). It is also acknowledged that the services described may have offered advice and support that extended beyond that included in the articles.

The diversity of terminology and a lack of clarity and consistency in the application of terms at times made categorisation challenging. For example, numerous terms were employed to describe FWA/S services [e.g., financial advice (38, 41), welfare and social prescription support service (23), social welfare advice (42), social welfare legal advice (42, 47)] and those who delivered them [e.g., link worker (34), housing professionals (24) specialist community link worker (25), money and welfare rights advice worker (37), advice worker (49)]. For clarity, these services will be referred to as specialist advisory services or FWA/S services, and those delivering them will be referred to as specialist advisors.

### Models of FWA/S within social prescribing

**Figure 3:**
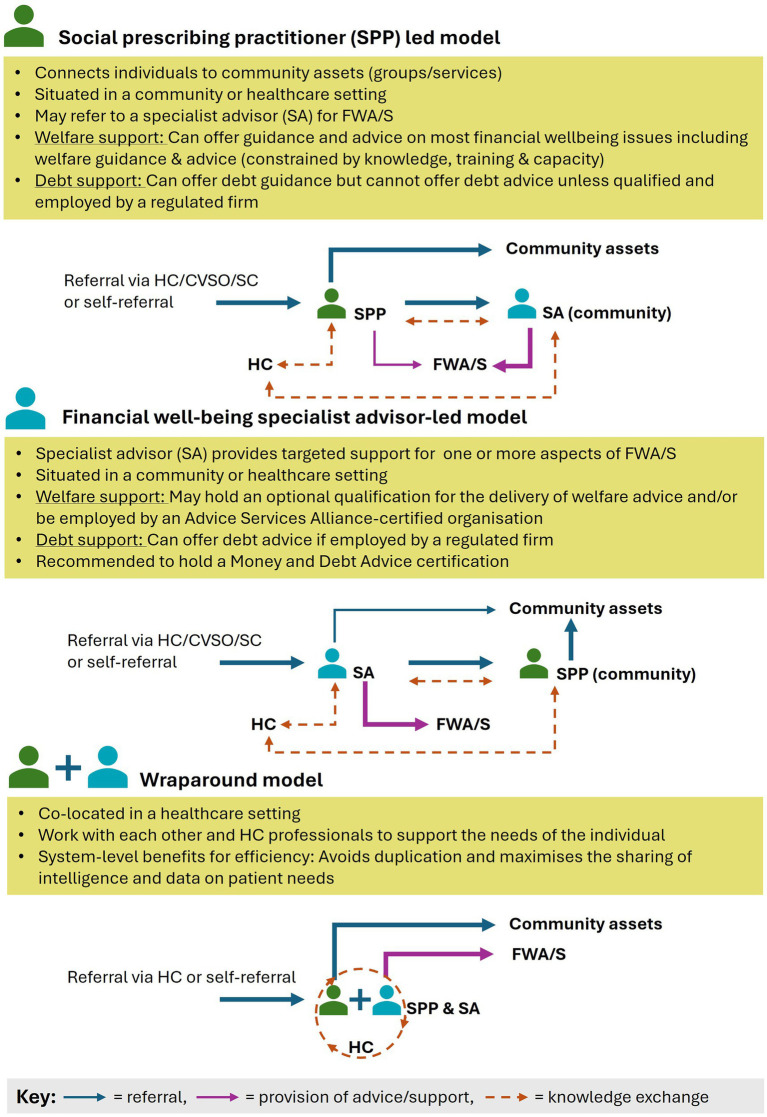
Models of FWA/S in social prescribing.

Three models of FWA/S within social prescribing were observed: the social prescribing practitioner (SPP)-led model, the specialist advisor-led model, and the wraparound model (see Figure 3).

In the SPP-led model, the individual accesses the SPP via referral from healthcare, social care, community and voluntary sector organisations, or self-referral. The SPP is situated in either a healthcare or community setting, and their primary focus is to support the individual by connecting them to community assets. Consequently, the SPP will likely make an onward referral or signpost to an external specialist advisor for FWA/S, delivered outside of a healthcare setting. The SPP may provide some FWA/S themselves, but this will be limited by their knowledge, training and capacity. To facilitate support for the individual, there may be some knowledge exchange between the SPP, healthcare professionals and the specialist advisor.

In the specialist advisor-led model, the individual accesses the specialist advisor via referral from healthcare, social care, a community and voluntary sector organisation or self-referral. The specialist advisor is situated in either a healthcare or community setting. Their primary focus is to provide the individual with targeted advice and support that includes, but may not be limited to, at least one of the elements of FWA/S. The specialist advisor may also refer the individual to a SPP or directly refer them to community assets. However, this will be constrained by the boundaries of their role, their knowledge of local community assets and their capacity. To facilitate support for the individual, there may be some degree of knowledge exchange between the specialist advisor, healthcare professionals and the SPP.

In the wraparound model, the SPP and the specialist advisor are both co-located within a healthcare setting. Access is usually via referral from healthcare or self-referral. A referral for FWA/S may pass directly to the specialist advisor, or it may initially be passed to the SPP. Unlike the other models, the SPP only provides support with accessing community assets, and the specialist advisor only provides FWA/S. Internal referral processes support this approach. As with the other models, knowledge exchange may occur between the SPP, specialist advisor and healthcare professionals. Such exchange may be facilitated by their increased proximity, and established protocols and procedures for sharing relevant patient intelligence and data.

### Social prescribing practitioner-led models of FWA/S

The review found only three articles that described the SPP-led model of FWA/S (31, 34, 35), all of which described the same Ways to Wellness intervention. The service was targeted at individuals aged 40–75 years with long-term conditions. SPPs were located in GP surgeries and the community, and referral pathways into the service were via GPs and practice staff, healthcare professionals, and staff from community and voluntary service organisations.

The SPPs aimed to facilitate connection with groups and services in their local community, including specialist advisors for FWA/S. In practice, however, they encountered several barriers to the delivery of such support. Some individuals referred into the service were already at crisis-point at the time of referral, presenting with complex physical and mental health needs in conjunction with multiple financial and social issues (34). These individuals required their financial and social issues to be addressed before the SPPs could focus on connecting them to other activities and services in the community (31, 34). However, insufficient availability of specialist advisory services in the community put the SPPs in a position where they often had to take practical action to support individuals with welfare benefits, debt and tenancy issues (31, 34, 35). In such instances, SPPs often felt they lacked the expertise to offer individuals the high-intensity, specialist support they required. Over time, the provision of such support placed them under increasing strain, as they dealt with increasing caseloads and the intensity of their clients’ needs (34).

### Specialist advisor-led models of FWA/S

24 articles reported on specialist advisor-led models of FWA/S. The most commonly reported specialist advisory service was the Citizens Advice Bureau (CAB), which appeared in fourteen articles (23, 27, 30, 36, 38–40, 42, 45–48, 50, 51). However, the breadth of support offered by the CAB was not uniform across articles, with some articles only describing support for welfare benefits and/or debt issues, while others included support and advice for employment, housing, health and care, and relationships. Other service providers included: Warm Wales (41), Energy Systems Catapult Ltd. (43, 44), the Cyrenians (25), National Debtline (33), the Greater Easterhouse Money Advice Project (GEMAP) (29, 49), Macmillan Cancer Support (32, 38). Four articles did not specify the service providers of the FWA/S they described (26, 28, 40, 51).

### Specialist advisor-led FWA/S targeting welfare benefit, debt and legal issues

Nineteen articles described interventions that utilised specialist advisor-led models to deliver targeted provision of FWA/S for welfare benefits and/or debt and/or legal issues (Supplementary Table 2).

Only three articles reported on FWA/S interventions targeted at welfare benefit, debt and legal issues that were delivered solely outside of healthcare settings: One study reported on a debt advice service for Jobcentre attendees with ongoing debt problems, which was delivered via the phone by National Debtline (33). The study found no significant difference in the rate at which intervention and control group respondents had resolved their debt problems at follow-up. In the second study, community nurses identified individuals ≥ 64 years who might benefit from a welfare benefits screening service and referred them to a specialist advisor, who then performed a home visit (29). The study demonstrated efficacy as a method of income maximisation, with application for areas of deprivation (29). The final report provided a case study of a CAB service delivered through organisations in the community (e.g., youth services, Barnardos and Jobcenters) and targeted at individuals aged 16–25 (40). The authors reported that findings suggest young people are generally underserved by advice services. However, access to the CAB service was associated with increased income, increased confidence in dealing with their financial issues and reduced stress (40).

Sixteen articles reported on services that were co-located in healthcare settings and utilised specialist advisor-led models to deliver FWA/S targeting welfare benefit, debt and legal issues (22, 23, 26, 27, 29, 30, 32, 36–39, 42, 45, 46, 48, 49). Referrals to specialist advisors predominantly came from GPs and practice staff. Several services also had pathways from allied health professionals, social care staff, and community and voluntary sector organisations (CVSOs) and via self-referral. Eleven articles did not detail any eligibility requirements for access to the specialist advisory services (22, 23, 26–28, 30, 37, 38, 42, 45, 47, 49). From context, however, it appears that this is likely due to the employment of a general open-door approach rather than an absence of detail on the author’s part. A general practitioner’s surgery was the most common healthcare setting (*n* = 14), although several services (*n* = 6) were also delivered in a hospital, secondary care and/or community settings. Only two articles reported on the co-location of interventions solely in hospitals; One reported on a service that provided FWA/S for welfare benefits, debt and housing/homelessness issues for individuals with complex mental health needs, who were screened and referred into the service on admission (48), and one study that provided a retrospective analysis of medical-legal partnerships (28).

#### Patient and staff perceptions of co-located specialist advisor-led FWA/S targeting welfare benefit, debt and legal issues

A number of articles reported that the co-location of specialist advisors within healthcare settings improved FWA/S access for vulnerable and deprived populations (30, 36–39, 42, 45, 46, 49), and helped capture individuals who would not have otherwise sought help (36, 37, 42, 45, 46, 49). For example, one mixed methods evaluation found that only 26% of individuals who accessed the co-located CAB service had previously used a high street CAB service (46). Similarly, another report on a co-located CAB service found that 39% of the individuals who accessed the service said they would only use a service located in a GP’s surgery (45). In a study of eight FWA/S services co-located in healthcare settings, nearly all participants expressed a preference to see a specialist advisor located within a GP surgery, and almost half of the participants interviewed stated that they would not have sought help elsewhere if the service was not available (36). Another report on a co-located FWA/S service found that, despite its longstanding presence in the community, delivery from general practices substantially increased the reach of the service, with many individuals accessing the service for the first time (49).

Qualitative data indicate that a preference for access to a specialist advisor in a local GP surgery was related to ease of accessibility, familiarity with the location, reduced stigma, the trusted nature of the provider, and the quality of the service and practical support they received (36, 37, 39, 42, 45, 46, 49). The GP’s surgery was considered to be easier to access, in general and for those with mobility issues. Booked appointments meant that individuals did not have to wait around, as they would with high street drop-in services (36, 39, 42, 45, 46), which could be especially uncomfortable or even impossible for someone with a severe disability. Additionally, the appointment-based system also allowed patients to ‘double up’ appointments with their primary health care professional and the specialist adviser (46). The familiarity of the GP surgery and referral to the service by a trusted member of the GP staff reduced anxiety and feelings of stigma around engagement with the service, supporting positive engagement between the specialist advisor and the patient (36, 37, 42, 45, 49).

Healthcare staff reported that co-located advisory services provided them with a referral pathway and a useful resource for patients presenting with financial well-being issues (22, 30, 37–39, 49). They felt that the specialist advisors helped them manage non-clinical demands and reduced the time they spent on non-health issues, especially if they provided direct support with aspects such as form-filling. This allowed them to spend their time more productively with patients, increasing job satisfaction (22, 27, 30, 37, 46). However, only one study recorded a reduction in the number of GP appointments following engagement with advice services (30). The presence of specialist advisors within a healthcare setting also improved staff’s knowledge and understanding of the welfare system and the socioeconomic problems their patients faced, allowing them to further contextualise their patients’ healthcare requirements (37, 38).

Interviews with staff identified several factors that supported the integration and delivery of FWA/S in healthcare settings (22, 37, 38, 42, 49): (1) Offering booked appointments; (2) Providing advice on a broad range of issues that are responsive to local needs; (3) Utilising the existing experiences, expertise and knowledge of practitioners; (4) Ensuring that specialist advisors are incorporated into the healthcare team (e.g., invited to staff meetings); and (5) Ensuring specialist advisors have access to medical records. Such access provided the specialist advisors with a comprehensive overview of the patients’ circumstances, supporting collaborative working with the GPs, reducing application processing time, appeals and ongoing casework (37, 49). These translated into financial gains for the patients, as demonstrated by one study which compared sites where specialist advisors did and did not have access to medical records (52).

#### Financial outputs from specialist advisor-led FWA/S interventions targeting welfare benefit, debt and legal issues

Eleven articles reported financial figures for specialist advisor-led FWA/S interventions targeting welfare benefit, debt and legal issues (Table 4), of which eight were co-located in healthcare settings. Nine articles reported increased income as a result of the services (27–29, 36, 40, 45, 46, 48, 49). Differences in how these were reported made direct comparisons between interventions difficult. Financial gain was reported as either income gained and/or managed debt and/or a combined figure for both. Additionally, some articles provided an average figure while others only provided a total figure, occasionally without any indication of the participant/patient number. Average income increases per individual ranged from £2689 (36) - £4,274 (48), total income gained per intervention from £118,111 (46) - £4,545,623 (27), and total debt written off ranged from £141,773.49 (46) - £7,660,593 (27). Four articles included an economic evaluation of the interventions (23, 27, 36, 37), although the use of different metrics again made direct comparisons difficult. For every £1 invested, there was an estimated gain of £39 in socioeconomic benefit (37), £3.40 - £4.69 SROI return (23), a financial return of £6.97 (27) – £15 (36), or £11.75 of managed debt (36).

**Table 4:** Provision of targeted FWA/S for welfare benefit, debt and legal issues.

# of articles	References	FWA/S			
		Welfare benefits	Debt	Legal	Housing/homelessness
5	(23, 26, 29, 30, 45)	**⚫**			
5	(22, 36, 37, 40, 49)	**⚫**	**⚫**		
1	(32)	**⚫**		**⚫**	
5	(27, 38, 39, 42, 46)	**⚫**	**⚫**	**⚫**	
1	(33)		**⚫**		
1	(28)		**⚫**	**⚫**	
1	(48)	**⚫**		**⚫**	**⚫**

#### The impact of specialist advisor-led FWA/S targeting welfare benefit, debt and legal issues on health and well-being

The evidence for improvements in mental and physical health as a result of accessing of specialist advisor-led FWA/S is far from robust. Several articles reported on improvements in social functioning, well-being, and quality of life (32, 33, 39, 40, 50), which were attributed to reductions in anxiety and stress, improved self-respect, choice and psychological well-being, and increased financial literacy (33, 39, 40). Patients also reported that accessing FWA/S gave them a more optimistic outlook for the future (49), improving their confidence and reducing their stress around addressing financial issues (40). However, while 78% of participants in one study reported feeling less anxious after accessing a specialist advisor, no significant improvements in physical or mental health were observed as a result of accessing the service (46). Similarly, an evaluation of a co-located welfare advice service reported a small but significant reduction in the prescribing of hypnotics and anxiolytics in patients after referral to the service, compared with the period before referral, but no change in appointments or referrals for mental health problems (30). There was some evidence to indicate that increasing income as a direct result of FWA/S is required for significant improvements in mental health and well-being to manifest in the short-term (36), but no evidence that such improvements persisted in the long-term (26). Overall, the review indicated a lack of robust evidence for improvements to health and well-being as a result of accessing FWA/S, rather than evidence to the contrary. This was especially true for changes to physical health and well-being, for which there were no significant improvements in any of the articles examined.

### Specialist advisor-led FWA/S targeting housing and homelessness

Three articles one grey literature report (48) and two studies (24, 25) focused on the provision of housing and homelessness support by specialist advisors. All three services were located in hospital settings, where specialist advisors can provide practical support with aspects such as searching for and bidding on properties, completing applications for health and medical rehousing, addressing rent arrears, organising a house clean, arranging an occupational therapist visit, and supporting people to stay within their current accommodation (24, 25). Findings indicate that such services can help support secure tenancy, resolve complex housing problems like eviction or repossession, facilitate earlier discharges, and prevent inappropriate discharges onto the streets and other unsuitable environments (24, 48).

The articles found that the specialist advisors acted as a point of in-hospital contact regarding discharge or ongoing care. They liaised with patients and members of the care team, and facilitated communication between different partners, service users, health and social work professionals, and family members (24, 25). However, it was identified that referral must occur close to admission to provide the specialist advisor with sufficient time to address issues. For example, patients who are experiencing homelessness but have been awarded temporary accommodation risk losing that accommodation if they miss their evening curfew or their housing manager is not made aware that they have been admitted to hospital (25). Hospital staff found that the incorporation of a specialist advisor into their multidisciplinary team helped reduce tension between different professional groups, allowed them to concentrate on clinical tasks, and was essential to ensure that the patient’s housing situation and wider needs were addressed at the earliest opportunity (24, 25). The support provided by the advisors, therefore, allowed for more informed decision-making between the hospital and community homelessness and housing services, and prevented loss of previously retained accommodation by ensuring that housing services and/or landlords were made aware of hospital admissions (25).

### Specialist advisor-led FWA/S targeting fuel poverty

Three articles focused on the provision of FWA/S targeting fuel poverty (41, 43, 44). The articles reported on interventions that: (1) Supported income maximisation through relieving fuel debt, help with bills/tariff switches, and benefits checks; and/or (2) The installation or repair of heating systems, draught proofing, and energy use advice. One article reported on the Warm Wales initiative (41), which seeks to integrate energy advice, support, and education with well-being and social prescribing to enhance health outcomes by addressing the root causes of fuel poverty. While total household savings of over £2million and a social impact value of £271 were reported (January 2024–September 2024), information relating to the accessibility and delivery of the service was relatively limited. Two reports on a Warm Home Prescription (43, 44), delivered by Energy Systems Catapult Ltd., collectively outlined a staggered approach to FWA/S for fuel poverty that began with an initial referral from a healthcare professional to an energy advisor for the provision of credit onto an energy account. The credit delivered the immediate impact of a warm home and highlighted to the recipient the importance of achieving healthy temperatures. This then opened the door to considering the implementation of longer-term solutions of warmth via energy efficiency and low-carbon heating improvements. The intervention supported the maintenance of a household temperature that did not put health at risk, and demonstrated £5.1 of well-being social value to patients for every £1 of project expenditure (44). Overall, there was limited evidence of a positive impact on mental and physical well-being (41, 43, 44). However, it was noted that individuals vulnerable to fuel poverty are often subject to a multitude of other factors impairing their health and home conditions, which may dilute the potentially beneficial effects of interventions that only target fuel poverty (41).

### Specialist advisor-led FWA/S targeting food poverty and employment

No articles focused on specialist advisor-led FWA/S interventions targeting either food poverty or employment issues. During the scoping process, several reviews and studies were identified that explored interventions that sought to provide support for food poverty and employment. However, these did not fall within the broad context of social prescribing as there were no indications of a referral process into and/or out of the intervention and/or a what matters conversation.

Ten of the articles included in this review described services that provided additional employment support (see Supplementary Table 1), although most did not detail the nature of this support. Those which did, described support with writing CVs (31), employment rights (42, 46), and representation at employment tribunals (42). We did not identify any studies that specifically examined the provision of employment support by social prescribing practitioner-type roles. The review only found two articles in which additional support for food poverty was mentioned: The independent review of Warm Wales reported the provision of food bank vouchers and onward referral for support from food banks and healthy food initiatives (41); and the Deep End advice worker project briefly outlined onward referral for support with various issues, including homelessness, food and fuel poverty (49).

#### Wraparound models of FWA/S

Only one report (47) examined wraparound models of social prescribing and FWA/S (47). The report outlines how the creation of a wraparound service that addresses social determinants of health in conjunction with physical and mental health needs offers system-level benefits for efficiency (avoiding duplication and maximising sharing of intelligence and data on patient needs) (47). The authors suggest that such systems also increase the likelihood that the patient’s needs will be met more promptly and cohesively and facilitate the proactive identification of issues and challenges, potentially before they become detrimental to the patients’ health and well-being.

However, findings from the report indicate that collaborative working between specialist advice services and social prescribing services is currently fairly limited, even when operating in the same space. Insecure funding and operating procedures that reinforce this level of separation can prevent some of the benefits of joined-up working from being realised. Recommendations to support the development of wraparound models of social prescribing and FWA/S include: (1) Developing a toolkit of guidance for the management of FWA/S in a healthcare setting, as well as guidance for social prescribing managers on effective service integration with specialist advisory services; (2) Considering, at the policy level, how the funding and status of FWA/S advice can be integrated into wider health and well-being agendas. Potentially via broadening the definition of care and working with partners at multiple levels, including Health and Well-being Boards and Integrated Care Systems; (3) Developing/including broader routine screening questions for patients aimed at triggering FWA/S referrals; and (4) Establishing automatic referral reminders within patient data management systems to trigger clinician referrals to FWA/S services and social prescribing (47).

## Discussion

The holistic concept of financial well-being extends beyond income and includes an individual’s sense of control over meeting both short-term and long-term financial needs (9). Social prescribing provides one possible means of supporting individuals to improve their financial well-being by supporting access to FWA/S. This review set out to scope the literature related to FWA/S in the context of social prescribing in the UK and RoI. From the literature, we identified three models of FWA/S within social prescribing: the SPP-led model, the specialist advisor-led model, and the wraparound model. The review found a diversity of terminology and services associated with FWA/S that imbues a degree of ambiguity regarding the provision FWA/S in the context of social prescribing.

The SPP-led and wraparound models clearly sit under the umbrella of social prescribing. In the case of referral by a SPP to a specialist advisor service for FWA/S, the advisor and associated service could be considered community assets. In the case of direct referral (e.g., from a healthcare professional) or self-referral to a specialist advisor, the advisor and associated service could still be considered community assets within the social prescribing pathway. However, the specialist advisor may also be considered to sit under the umberall term of SPP, if they provide onward referral to other community assets and complete a what matters conversation.

The SPP-led model benefits from a single referral process into social prescribing, which may increase the likelihood of a referral happening in the first place. However, for patients, this multi-referral process can be disheartening, and long wait times to see a specialist advisor for FWA/S advisor can undermine perceptions of the effectiveness of social prescribing for both patients and practitioners (42, 47). The ubiquity of SPPs, in both healthcare and community settings, makes it likely that this is the most common model of FWA/S in social prescribing. It is therefore surprising that the review found only three articles that reported on this mode of FWA/S delivery, all relating to the same service (36, 39, 40). In these examples, the preference of the SPPs was to connect individuals to specialist advisory services in the community. However, the referral of service users in a state of crisis and a lack of available services within the community necessitated their direct provision of FWA/S, placing them under considerable strain (31, 34). It is not possible to ascertain from the findings of this review just how commonplace onward referral from a SPP to a specialist advisor for FWA/S is, but it does appear to be under-reported in the literature. It is also not possible to ascertain if the delivery of FWA/S by SPPs is commonplace. However, research indicates that referrals to specialist advisory services for FWA/S services far exceed availability (51), suggesting both a likelihood that SPPs are providing FWA/S out of necessity and a need to increase the provision of specialist advisors.

The largest proportion of articles related to services that sat within the specialist advisor-led model of FWA/S, and which were predominantly co-located within healthcare settings. The articles reported that such co-located services had positive outcomes for patients, capturing individuals who would not have otherwise sought advice from services in the community and allowing them to address their FWA/S issues promptly (36, 39, 42, 45, 46). Co-located specialist advisors also increased staff’s job satisfaction and understanding of how a patient’s socioeconomic challenges were impacting their well-being [e.g., (22, 39, 49)]. Staff reported that the efficacy of such co-located services could be positively impacted by granting the specialist advisory services access to medical records (22, 37, 49) and integrating advisors into the healthcare team (22, 37, 49).

Given the reported benefits of the specialist-advisor-led model of FWA/S, it is perhaps surprising that the review found only one article related to the wraparound model (47), which has the potential to be even more efficacious. The model lends itself to addressing the social determinants of health in conjunction with physical and mental health needs, through system-level benefits that are supported by increased proximity and protocols and procedures for sharing relevant patient intelligence and data (47). However, the article reported that findings indicate that insecure funding and operating procedures that reinforce separation are negatively impacting the level of collaborative working between specialist advice services and social prescribing services (47). Barriers that likely extend to, and are perhaps even more pronounced in, the other models of FWA/S.

The review captured several articles that reported on FWA/S services within hospital settings aimed at supporting individuals experiencing housing and homelessness issues (24, 25, 48). Patients who experience homelessness experience a high frequency of hospital readmission after discharge, often for the same or similar reasons for initial hospitalisation (53). The articles highlighted the potential of such services to save health and social care services time, money and resources by reducing inappropriate hospital discharge, readmission and stay duration (24, 25, 48). These services may be especially beneficial for hospital patients who have mental health issues, as they are more likely to experience problems with debt, housing, homelessness and accessing welfare benefits, in part because repeated hospital admissions destabilise their housing situation (24, 48). FWA/S may, therefore, also reduce the risk of them experiencing a mental relapse by directly acting on the immediate cause(s) of acute stress (48).

Several articles reported that FWA/S services positively impacted well-being, and quality of life (33, 39, 40, 51), primarily through the provision of choice and control and reductions in associated stress and anxiety. As with previous reviews (17, 54, 59), we found the evidence for such an effect to be mixed. Overall, the review indicated a lack of robust evidence for improvements to mental and physical health and well-being as a result of accessing FWA/S, rather than evidence to the contrary. Such findings may, at least in part, be due to the methodological approaches utilised. Few studies used standardised measures, relying instead on qualitative data and non-standardised questionnaires and surveys. Even fewer employed measures that examined changes in physical health. However, it should also be considered that a longer duration is required to observe a significant impact of FWA/S physical health, than that required to observe changes in mental health and well-being. Non-significant improvements in health should also not be readily dismissed. As one article noted, even modest improvements in physical health can make a significant contribution to a patient’s perception of their quality of life (26).

### Implications for policy and practice

Policy has aspirations for utilising social prescribing to help address the social determinants of health (11, 52, 56, 57), which includes supporting financial well-being. However, greater consideration is required at the policy level of how the funding and status of FWA/S advice can be integrated into wider health and well-being agendas. Additionally, more robust evidence and guidance are required for the different models of FWA/S in social prescribing to ensure the efficacious direction of time and resources, which may differ across nations and regions. Many of the studies examined in this review employed bespoke questionnaires rather than standardised measures, making direct comparison between services challenging. The development of guidance, for example, in the form of Welsh Governments core data (11) set may assist with this in the future.

One possible means of increasing the availability of FWA/S is by SPPs undertaking training to extend their role (13). This review did not find any evidence that this was happening in practice or provide insight into SPPs appetite for such a change. An increased offering of support would align with policy objectives that aim to reduce the impact of poverty and inequality on health and well-being, e.g., (58). However, asset mapping and developing working relationships to effectively connect individuals to the community takes considerable time and knowledge. As highlighted by several of the studies in this review (31, 34, 35), there is a danger that extending the SPP role may negatively impact the efficacy of social prescribing services without achieving the outcomes associated with engagement with a specialist advisor. Additionally, the review did not provide any indication of SPPs current level of awareness of the regulations and recommendations that surround the provision of different elements of FWA/S. Clearer guidance is needed to ensure that SPPs do not inadvertently end up providing support that exceeds their professional boundaries.

Both specialist advisor-led models and wraparound models highlight system-level benefits of co-location in healthcare settings for addressing health and financial well-being (37, 47, 49). However, all models require more secure funding to ensure the suitable provision of specialist advisors in both healthcare and community settings, and to ensure social prescribing and specialist advisory services have the longevity and security required to support collaborative working. Support and guidance are also required for the efficacious establishment of protocols and procedures that support data and information sharing (37, 47, 49), as well as the routine incorporation of systems that support self-initiated referrals (41), and screening questions and automatic reminders that trigger FWA/S referrals (47).

## Limitations

The review is subject to several limitations. Firstly, direct comparison between articles and interpretation of findings were hindered by several challenges. There was a lack of consistency in the reporting of the included articles. Many articles failed to report sufficient detail on aspects such as the support offered by services, service providers, eligibility and referral information, number of participants/patients, and assessment and/or outcome measures. Additionally, there were some discrepancies and ambiguity in the application of terminology surrounding FWA/S.

As with any review, we had to balance feasibility and practicality with aspiration. Grey literature was sourced from citation searches and requests to stakeholders. We did not perform a Google (or similar) search, as exploratory searches indicated that we would have been inundated with non-relevant articles. Additionally, we were unable to access the full text of 11 grey literature articles, all published prior to 2015. There is some evidence to indicate a bias of healthcare-related literature in peer-reviewed journals (59). Our grey literature restrictions may, therefore, have contributed to a bias within this review of literature related to healthcare-based specialist advisors.

The review suggests a potential gap in the literature regarding FWA/S for food poverty, fuel poverty and employment, within the social prescribing sphere. However, it is acknowledged that evidence of this support may exist predominantly within grey literature and/or was reported in a manner that was outside of the scope of this review. For example, several articles were identified during the scoping process that examined the benefits of interventions for food poverty and fuel poverty. However, these did not report on crucial aspects that allowed for their inclusion under the broad umbrella of social prescribing, i.e., someone in a SPP-type role, a referral process into and/or out of the service, or a what matters conversation.

A scoping review was an appropriate methodological choice to identify the extent and nature of available evidence relating to FWA/S in the context of social prescribing within the UK and RoI, as well as the gaps in knowledge within the field (18, 19). However, it is beyond the requirements of such a review to assess the quality and rigour of the included literature, limiting our ability to make inferences of effectiveness.

## Conclusion

Policy and guidance demonstrate enthusiasm for utilising social prescribing to support the delivery of FWA/S. The delivery of FWA/S by specialist advisors is relatively well documented, especially within healthcare settings. The review highlighted benefits of such support for both staff and patients, as well as benefits of wraparound services that co-locate specialist advisors and SPPs within healthcare settings. However, despite a need for specialist FWA/S services, budget cuts have reduced the availability of these services, with potential implications for SPPs who may be forced to provide direct provision themselves. Currently, little is known regarding SPPs current levels of referral to specialist advisors or their direct provision of FWA/S. Additionally, the review does not shed light on whether SPPs have the desire to undertake additional training to be able to adopt the provision of FWA/S into their role, which has the potential to be time-consuming and capacity-reducing. Future changes to policy and practice should first consider the additional research required to ensure the efficacious employment of funding and delivery of FWA/S, which may differ across nations and regions.

## Appendix

Glossary

**Advice:** Involves recommending a specific course of action for a person (13).

**Community assets:** A collective term for anything that can be used to improve the quality of community life. This can include community groups, interventions and services, as well as buildings, land, or even a person within a community.

**Debt advice:** The recommendation of a specific course of action, in relation to an individual’s debts, based on their circumstances, goals and information provided. Advice must be provided by a qualified individual, and it is recommended that they hold a Money and Debt Advice certification in Money Advice Practice (via the Institute of Money Advisors or Chartered Institute of Credit Management). Debt advisors must work for an organisation that is appropriately insured and authorised by the Financial Conduct Authority (13, 20).

**Financial advice:** The provision of advice on aspects such as retirement and pensions, debt, taxes, protecting assets, investments and savings by a qualified and regulated individual or online by a regulated organisation (13, 20).

**Financial well-being advice and support (FWA/S):** The provision of guidance and/or advice and/or practical support with welfare benefits, debt management, fuel poverty, food poverty, employment, tenancy issues and avoiding homelessness, and legal issues associated with the aforementioned aspects of FWA/S. There are different requirements and recommendations associated with providing guidance, advice and practical support in the different areas that fall within FWA/S.

**Guidance:** The provision of information, but without recommending a course of action. This can range from signposting to providing technical information for issues such as welfare benefits, debt and budgeting (13).

**Practical support:** The provision of practical assistance with aspects such as completing welfare benefit or tenancy applications.

**Social prescribing:** An umbrella term that describes a person-centred approach to connecting people to local community assets. It can help empower individuals to recognise their own needs, strengths, and personal assets and to connect with their own communities for support with their personal health and well-being.

**Social prescribing practitioner (SPP):** An umbrella term to describe someone who assists individuals with identifying their non-medical needs through a what matters conversation, and who helps them co-produce an action plan and access local community assets, such as groups, interventions or services. If they identify a potential medical need, they may also refer the individual to a GP or pharmacist. SPPs can deliver welfare benefits advice, but it is good practice for them to have been trained to either guidance or advice level and be acting within their professional boundaries (13, 20). SPPs can provide debt guidance but not debt advice unless they are appropriately qualified and employed by an organisation that is authorised by the Financial Conduct Authority (13, 20).

**Social-welfare-legal advice:** The provision of advice relating to welfare benefits, debt, housing, energy, family, employment, education, immigration and consumer law (13).

**Specialist advisor:** In the context of FWA/S, a specialist advisor is anyone who: (1) is not employed as a social prescribing practitioner, where the primary focus of support is to connect individuals to community assets; and (2) provides targeted guidance, advice and/or support for at least one of the elements of financial well-being.

**Welfare benefits advice:** The provision of advice and support to help an individual identify any welfare benefits or other support offers that they are eligible for. Support can also be provided for current welfare benefit claims. This advice is not regulated by an external body, but organisations have the option of being monitored and achieving the Advice Quality Standard, which is the recognised quality mark in England and Wales. While technically not required, advisors can complete a qualification, and it is good practice for those providing welfare benefits advice to have been trained to either guidance or advice level and be acting within their professional boundaries (13, 20).

**Welfare support referral:** The referral of individuals to organisations that offer social welfare, legal and financial support. Welfare support referral can help individuals to address issues which can directly impact upon their health, well-being and quality of life. Examples of welfare support referral services and activities include debt management, reporting violations of housing standards, avoiding homelessness, employment or education/training, access to healthcare, and engagement with interventions from social services. Advice services may be located together with, or independently from, health services.
