# Supplementary material for: Financial well-being advice delivered within the context of social prescribing in the UK and the Republic of Ireland
Source: Front Public Health. 2026 May 29;14:1789734. doi: 10.3389/fpubh.2026.1789734 (PMC13260262; doi:10.3389/fpubh.2026.1789734)
Supplement: Supplementary file 1 [file Table_1.pdf]

Supplementary Table 1. Delivery and eligibility of FWA/S advice and support

Title	Delivered by Specialist Advisor	Intervention setting	Service provider	Eligibility	Referral into service	Type of advice/support							
						Welfare benefits	Debt	Legal	Employ.	Housing/ homelss.	Fuel poverty	Food poverty	Onward referral
Link Worker social prescribing to improve health and well-being for people with long-term conditions: qualitative study of service user perceptions (32)	No:<br>General SP	Not specified	Ways to Wellness	Aged 40–74 years with one or more LTCs	GPs and practice staff	○	○	○	○				●
Link workers' perspectives on factors enabling and preventing client engagement with social prescribing (35)	No:<br>General SP	Not fixed:<br>GP surgeries, community and home visits.	Ways to Wellness	Aged 40–74 years with one or more LTCs	HC staff	○			○	○			●
Service-users' perspectives of link worker social prescribing: a qualitative follow-up study (36)	No:<br>General SP	Not fixed:<br>GP surgeries, community and home visits.	Ways to Wellness	Aged 40–74 years with one or more LTCs	HC & CVSO staff	○	○		○				●
Examining the service offering provided by Warm Wales for communities across Wales (42)	Yes	Community based	Warm Wales	Not specified	Network partners<br>Engagement officers<br>Self-referral	○	○	○			●	○	○
Warm Home Prescription® Insights and Impact Report (44)	Yes:<br>Instillations manager	Phone call<br>Home visit	Energy Systems Catapult Limited	Health conditions exacerbated by the cold	HCPs<br>SPPs						●		
Warm Home Prescription Impact and Value for Money Report (45)	Yes<br>Energy adviser	Phone call<br>Home visit	Energy Systems Catapult Limited	Patients who cannot afford the heating they need to stay well.	HCPs<br>SPPs						●		
Reducing hospital readmissions amongst people experiencing homelessness: a mixed methods evaluation of a multi-disciplinary hospital in-reach programme (26)	Yes	Hospitals	The Cyrenians	Patients at risk of homelessness on admission	Cyrenians staff<br>Primary and secondary HCPs	○				●			
Exploring the Impact of a Housing Support Service on Hospital Discharge: A Mixed-Methods Process Evaluation in Two UK Hospital Trusts (25)	Yes<br>Housing support coordinators	General and MH hospitals	Not specified	Patients without appropriate housing	HCPs	○				●			○
Changing Fortunes: Results from a Randomized Trial of the Offer of Debt Advice in England and Wales (34)	Yes	Phone call	National Debtline	18+ with ongoing debt problems	Targeted jobcentre attendees		●			○			○
Medical-legal partnerships: 11 years' experience of providing acute legal advice for critically ill patients and their families. (29)	Yes<br>Senior solicitor	Hospital	Legal firm (not specified)	Not specified	HCPs	○	●	●	○	○			
Welfare advice for people who use mental health services (49)	Yes:<br>Regulated	Hospitals	CAB	Complex mental health needs	Ward staff - Patients screened on admission	●	●	○		●			○

Impact of co-located welfare advice in healthcare settings: prospective quasi-experimental controlled study (37)	Yes	GP surgeries	CAB	18+	GPs and practice staff<br>Self-referral	●	●						
The Deep End Advice Worker Project: Embedding an Advice Worker in General Practice Settings (50)	Yes	GP surgeries	GEMAP: Greater Easterhouse Money Advice Project	Not specified	GPs and practice staff	●	●			○	○		○
Case Study: co-location of advice workers in medical practices in Dundee and Edinburgh (38)	Yes	GP surgeries	Granton-Information Centre	Not specified	GPs and practice staff	●	●						
Building a Healthier Wales Coordination Group. Progress to date (41)	Yes:<br>Pilot project - Regulated	Organisations such as youth services, Barnardos and Job Centres.	CAB	16-25 years	Not specified	●	●	○	○				
Co-located welfare advice in general practice: A realist qualitative study (23)	Yes	GP surgeries	Not specified beyond independent specialist welfare rights advisers	Not specified	GP practice staff<br>Self-referral	●	●		○				
Social Return on Investment (SROI) Evaluation of Citizens Advice on Prescription: A Whole-Systems Approach to Mitigating Poverty and Improving Wellbeing (24)	Yes:<br>Regulated	GP surgeries<br>Secondary care services	CAB	Not specified	GPs and practice staff<br>Secondary HCPs	●	○		○				○
Roll-out of a nurse-led welfare benefits screening service throughout the largest Local Health Care Co-operative in Glasgow: An evaluation study (30)	Yes<br>Money advice workers	Home visits	GEMAP: Greater Easterhouse Money Advice Project	64 + years with care needs	Community nurses	●							
Evaluation of welfare advice in primary care: effect on practice workload and prescribing for mental health (31)	Yes:<br>Regulated	GP surgeries	CAB	Not specified	GPs and practice staff	●							
Welfare Advice in General Practice - The Better Advice, Better Health Project in Wales (46)	Yes:<br>Regulated	GP surgeries and community	CAB	Not specified	GPs and HCPs<br>Self-referral	●							
What is the impact on individual health of services in general practice settings which offer welfare benefits advice? (27)	Yes	GP surgeries	Not specified	Not specified	Not specified	●							○
Addressing the Financial Consequences of Cancer: Qualitative Evaluation of a Welfare Rights Advice Service (33) (	Yes	GP surgeries and community	Macmillan Cancer Support	Cancer patient or carer	HC & CVSO staff<br>Self-referral	●		●					○
Citizens advice in primary care: a qualitative study of the views and experiences of service users and staff (28)	Yes:<br>Regulated	GP surgeries	CAB<br>*no specific focus of support	Not specified	GPs and practice staff<br>Self-referral	●	●	●					○
How Social Welfare Legal Advice and Social Prescribing can work collaboratively in healthcare settings (48)	Yes	GP surgeries, hospitals, community settings and home visits.	Examples from case studies:<br>CAB<br>Help on your doorstep	Not specified	GPs and HCPs<br>SPPs	●	●	●		○			○

Reducing health inequities in London by improving access to social welfare advice through greater collaboration between the healthcare, local authority and advice sectors. Full Report (43)	Yes<br>Multiple examples	Hospitals<br>GP surgeries and community	Social welfare advice agencies (example includes CAB)	Not specified	HC and SC staff<br>SPPs<br>Self-referral in some settings	●	●	●	○	○			
Liverpool Citizens Advice on Prescription Interim evaluation report - January 2023 (40)	Yes	GP surgeries<br>Secondary care services	CAB	At risk of financial and/or social hardship	GP practice staff<br>Secondary HCPs<br>Third-sector mental health organisations<br>Self-referral<br>CVSOs	●	●	●	○	○	○		○
The Health Justice Landscape in England & Wales. Social welfare legal services in health setting (39)	Yes	GP surgeries, hospitals, MH services, care homes and community settings	Examples include CAB and Macmillan Cancer Support.	Not specified	GP practice staff,<br>HCPs and AHPs<br>Onward referral from SPPs	●	●	●		○			○
Evaluation of the Warrington district CAB GP outreach project (47)	Yes	GP surgeries	CAB	At risk of financial and/or social hardship	GP practice staff<br>HCPs and AHPs<br>Self-referral	●	●	●	○	○			

KEY: SPP = social prescribing practitioner, AHP = allied health professional, HCP = healthcare professional, CAB = Citizens Advice Bureau, ● = primary form of advice/support, ○ = secondary form of advice/support, Employ. = employment, Homelss = homelessness
