# Supplementary material for: Financial well-being advice delivered within the context of social prescribing in the UK and the Republic of Ireland
Source: Front Public Health. 2026 May 29;14:1789734. doi: 10.3389/fpubh.2026.1789734 (PMC13260262; doi:10.3389/fpubh.2026.1789734)
Supplement: Supplementary file 2 [file Table_2.pdf]

Supplementary Table 2. Financial outputs from specialist advisor-led FWA/S targeting welfare benefit, debt and legal issues

Title	Economic evaluation	Financial gain
Citizens advice in primary care: a qualitative study of the views and experiences of service users and staff (28)	£1 invested in the project, it secured £6.97 in additional income for its users and managed £11.75 of their debts.	Data collected for 2009-2010 indicate that of 3490 clients seen, one in four (27%) received additional income as a result of advice. Total income = £4,545,623 and £7,660,593 of debt was managed.
Welfare advice for people who use mental health services (49)		N = 113: Average increased income = £4,274/year
Medical-legal partnerships: 11 years' experience of providing acute legal advice for critically ill patients and their families (29)		N = 346: Assisted with various financial or housing issues, including liaison with creditors or the mortgage company and the cancellation of various contracts without incurring a cancellation fee. Over £129,000 of patient debt written off by creditors.
Impact of co-located welfare advice in healthcare settings: prospective quasi-experimental controlled study (37)	Per capita participants received £15 per £1 invested into the service.	N = 204: Total additional income (managed debt, income increase and one-off payments) = £793,135 , average income increase = £2689
The Deep End Advice Worker Project: Embedding an Advice Worker in General Practice Settings (50)		N =174: Total increased income = £644, 819.10
CASE STUDY: Co-location of advice workers in medical practices in Dundee and Edinburgh (38)	Every £1 invested in the service generated around £39 in social and economic benefits.	
Social Return on Investment (SROI) Evaluation of Citizens Advice on Prescription: A Whole-Systems Approach to Mitigating Poverty and Improving Well-being (24)	The overall study reported a positive SROI return range of GBP 1: GBP 3.40–GBP 4.69.	
Roll-out of a nurse-led welfare benefits screening service throughout the largest Local Health Care Co-operative in Glasgow: An evaluation study (30)		£925,015 = total Attendance Allowance (n =348), £169,372.82 = total passport-related benefits plus their AA benefits (n = 73), £42,036.32 = total in other benefits for those who did not qualify for AA (n = 28)
Building a Healthier Wales Coordination Group. Progress to date (41)		N = 153: Total income gain of £409k + £105k other gain, including debt written off.
Welfare Advice in General Practice - The Better Advice, Better Health Project in Wales (46)		Total benefit claim = £3,448,672 (no participant/user number)
Evaluation of the Warrington district CAB GP outreach project (47)		N = 96: Total lump sum payments = £22,186.38, total annual gains = £118,111.86, Total debt written off = 141,773.49. Total gain by all clients as a result of contact with the CAB GP Outreach Project (August 2003 - September 2004) = £356,753.95
